# Biophysical properties of alveolar surfactant in drever dogs with hunting associated pulmonary edema

**DOI:** 10.1186/s13028-024-00745-x

**Published:** 2024-05-31

**Authors:** Sanna Johanna Viitanen, Sabrine Moya Gehani, Anni Maria Tilamaa, Minna Marjaana Rajamäki, Ruud Anthonius Wilhelmus Veldhuizen

**Affiliations:** 1 Department of Equine and Small Animal Medicine, Faculty of Veterinary Medicine, University of Helsinki, Koetilantie 2, 00790 Helsinki, Finland; 2 Departments of Medicine and Physiology & Pharmacology, University of Western Ontario and Lawson Health Research Institute, 800 Commissioners Road, London, ON Canada

**Keywords:** Canine, Non-cardiogenic pulmonary edema, Pulmonary surfactant activity, Respiratory

## Abstract

**Background:**

A syndrome of acute non-cardiogenic pulmonary edema associated with hunting is prevalent in the drever breed, but etiology of this syndrome is currently unknown. Alveolar surfactant has a critical role in preventing alveolar collapse and edema formation. The aim of this study was to investigate, whether the predisposition to hunting associated pulmonary edema in drever dogs is associated with impaired biophysical properties of alveolar surfactant. Seven privately owned drever dogs with recurrent hunting associated pulmonary edema and seven healthy control dogs of other breeds were included in the study. All affected dogs underwent thorough clinical examinations including echocardiography, laryngeal evaluation, bronchoscopy, and bronchoalveolar lavage (BAL) as well as head, neck and thoracic computed tomography imaging to rule out other cardiorespiratory diseases potentially causing the clinical signs. Alveolar surfactant was isolated from frozen, cell-free supernatants of BAL fluid and biophysical analysis of the samples was completed using a constrained sessile drop surfactometer. Statistical comparisons over consecutive compression expansion cycles were performed using repeated measures ANOVA and comparisons of single values between groups were analyzed using T-test.

**Results:**

There were no significant differences between groups in any of the biophysical outcomes of surfactant analysis. The critical function of surfactant, reducing the surface tension to low values upon compression, was similar between healthy dogs and affected drevers.

**Conclusions:**

The etiology of hunting associated pulmonary edema in drever dogs is not due to an underlying surfactant dysfunction.

**Supplementary Information:**

The online version contains supplementary material available at 10.1186/s13028-024-00745-x.

## Background

A syndrome of acute non-cardiogenic pulmonary edema associated with hunting has been described in dogs already in the 1970s [1]. The syndrome affects almost exclusively drevers, a Swedish chondrodystrophic hunting dog breed, but cases have also been documented in other breeds with similar body conformation and hunting style [2, 3]. Affected dogs typically develop clinical signs starting at a young age and signs usually reoccur repeatedly if dogs are allowed to hunt strenuously. Clinical signs begin during or within minutes after hunting and include tachypnea, coughing, dyspnea and lethargy [2, 4]. Thoracic radiographs reveal typically caudo-dorsal lung infiltrates, which clear without specific treatment and in echocardiography the left atrium-aorta ratio (LA/Ao) is normal [2]. Lung histopathology in dogs euthanized during dyspnea has confirmed pulmonary edema as the cause of clinical signs [2]. A study utilizing owner questionnaires to drever dams and sires in Sweden suggests that the condition may be a hereditary disease in the breed [4]. However, despite research efforts the etiology of this syndrome remains largely unknown.

In general, the literature on pulmonary edema identifies a variety of contributing factors since the air-blood barrier in lungs consists of the surfactant containing alveolar hypophase, the alveolar epithelium, the capillary endothelium and their basal laminas. Fluid balance is regulated by the Starling forces, an interplay between intravascular and interstitial hydrostatic and oncotic pressures. Pulmonary edema forms, when the pressure difference favors fluid shift from the vasculature to the interstitium or when vascular permeability increases [5]. A classic example is the formation of cardiogenic pulmonary edema, where vascular pressure increases due to left sided cardiac dysfunction. Moreover, variable mechanisms for non-cardiogenic pulmonary edema have been described including conditions causing increased vascular permeability, increased intrathoracic negative pressure due to upper airway obstruction and neurogenic mechanisms [5]. Interestingly, maximal exercise is also considered a condition favoring pulmonary edema formation and during maximal exercise the lung is subjected to substantial stressors increasing fluid shift from vasculature to the interstitium [6]. Pulmonary blood pressure increases, and large numbers of pulmonary capillaries are recruited, which enables an increased pressure and surface area for fluid diffusion [6]. In normal circumstances, the increased fluid shift into lung interstitium during exercise is balanced by markedly increased lymphatic flow preventing the development of clinical pulmonary edema [7–9].

In addition to vascular changes during exercise, the lung is also subjected to increased dynamic pressure and volume changes due to intensive ventilation. To protect the alveoli against damage due to pressure changes, the alveoli are stabilized with the alveolar surfactant system and the elastic connective tissue of the pulmonary interstitium [10]. The alveolar surfactant is produced by the alveolar type II epithelial cells, stored intracellularly in lamellar bodies and secreted into the alveolus [10]. Surfactant is composed of lipids (90%) and proteins (10%). The majority of surfactant lipids are phospholipids, and the most abundant, phosphatidylcholine, is present largely as desaturated dipalmitoylphosphatidylcholine, which is a major surface tension reducing component [11]. The protein fraction of surfactant consists mainly of the four surfactant associated proteins: Surfactant proteins A, B, C and D, which have both surface tension lowering and immunomodulatory properties [11]. Surfactant forms a surface film at the air–liquid interface and is responsible for the critical reduction of surface tension during ventilation thereby stabilizing the alveoli and protecting against end-expiratory collapse [10]. Low surface tension is also important in balancing pressures on the respiratory epithelium and thereby ensuring that net fluid flow is directed from the alveolar space into the interstitium, keeping the alveoli dry [11, 12]. Earlier experimental models have confirmed that loss of alveolar surfactant and the consequent increase in surface tension leads to the development of pulmonary edema [13–15].

Based on this critical role of surfactant in preventing edema, our hypothesis was that the etiology of hunting associated pulmonary edema in drever dogs is associated with impaired biophysical properties.

## Methods

### Study design and population

A prospective observational study was carried out. Privately owned drever dogs with recurrent hunting associated pulmonary edema examined at the Veterinary Teaching Hospital of the University of Helsinki between September 2022 and June 2023 were included in the study. Additionally, healthy dogs of other breeds with no history or clinical signs of respiratory disease were included as healthy controls.

The diagnosis of hunting associated pulmonary edema was made, when there were recurrent signs of respiratory distress (tachypnea or dyspnea with or without cough) associated with hunting starting at young age (first episode ≤ 6 years of age) and lack of other diagnosed cardiorespiratory diseases capable of causing the clinical signs [2]. Dogs receiving any medications were excluded from the study.

In affected drevers, patient data and thoracic radiographs obtained during previous episodes of hunting associated respiratory distress were requested with owner consent from the referring veterinarians and assessed by the authors. Additionally, video material obtained by the owners describing the clinical signs during respiratory distress was evaluated when available.

### Clinical examinations

All affected dogs were examined in between episodes of respiratory distress when they were clinically normal. The dogs underwent thorough clinical examinations to rule out underlying cardiorespiratory diseases potentially causing the signs. Venous blood samples were obtained for hematology (Advia 2120i, Siemens AG, Munchen, Germany) and serum biochemistry (Konelab 30i, Thermo Scientific, Vantaa, Finland). Fecal samples were collected on three consecutive days and examined with ZnSO_4_ flotation and Baermann sedimentation methods. Echocardiography (Philips iE33, Royal Philips Electronics, Amsterdam, Netherlands) was performed on all affected dogs prior to sedation by a cardiologist (MMR). Standard echocardiographic measurements from three consecutive cardiac cycles were measured and averaged [16].

Anesthesia was planned individually for each dog by a veterinary anesthesiologist. Dogs were premedicated with butorphanol (Butordol 10 mg/ml, Intervet International GmbH, Unterschleissheim, Germany) and either acepromazine (Plegicil 10 mg/ml, Bela-Pharm GmbH & Co KG, Germany) or dexmedetomidine (Dexdomitor 0.1 mg/ml, Orion Pharma, Espoo, Finland) and anesthesia was induced with intravenous propofol (Propovet Multidose 10 mg/ml, Fresenius Kabi AB, Uppsala, Sweden). Prior to intubation pharyngeal and laryngeal structures were evaluated, laryngeal function was assessed with or without doxapram-stimulation (Dopram 20 mg/ml, Haupt Pharma Wülfing GmbH, Gronau, Germany) and tracheoscopy was performed. General anesthesia was maintained after intubation with inhaled sevoflurane (Sevohale 100% v/v, Chanelle Pharmaceuticals Manufacturing Ltd., Galway, Ireland). Computed tomography (CT) imaging (head, neck and thorax) was performed (GE LightSpeed VCT 64, GE Healthcare, Fairfield, Connecticut) under general anesthesia during a ventilatory pause and CT images were assessed by a veterinary radiologist (AT). Bronchoscopy was performed after CT with a 4.9 mm flexible endoscope (Olympus GIF-N180, Olympus Medical Systems Europa GmbH, Hamburg, Germany) and samples for cytology and quantitative bacterial culture were obtained by weight-adjusted bronchoalveolar lavage (BAL) from right middle lung lobe and left caudal lung lobe [17]. Quantitative aerobic bacterial cultures were performed from bronchoalveolar lavage fluid (BALF) samples immediately after sampling as described previously [18]. Cytology slides were prepared with cytocentrifugation (Cytospin4, Thermo Scientific, Vantaa, Finland) and a differential cell count was calculated manually (500 cells) after May-Grünwald Giemsa staining. BALF samples were centrifuged at 1500 rpm for 10 min to separate cells from supernatant, which was frozen immediately after separation and stored at − 80 °C until analysis.

All healthy dogs participating in this study were admitted to the hospital for routine radiographic screening for hip dysplasia. Dogs were sedated with butorphanol and dexmedetomidine and blood samples were obtained for hematology and serum biochemistry as described previously. Bronchoscopy and BAL were performed after the radiography under short acting intravenous anesthesia using propofol and BALF samples were processed in a similar manner as in the affected dogs.

### Preparation of surfactant

Frozen cell-free supernatants from BAL fluids were utilized. After thawing, the fluid was centrifuged at 40,000 g for 15 min to obtain a surfactant pellet. This pellet was resuspended in a small volume (200–500 µl) of buffer (0.9% NaCl, 1.5 mM CaCl_2_, and 2.5 mM HEPES [pH 7.4]). An aliquot of the samples was obtained to perform a methanol-chloroform extraction, followed by a phosphorus and colorimetric assay to determine the phospholipid concentration [19, 20]. The surfactants were resuspended at 2 mg phospholipid/mL in same buffer.

### Measurement of minimum surface tension

Biophysical analysis of the surfactant samples was completed using a custom built constrained sessile drop surfactometer (CDS) and associated software [21]. Briefly, an 8 µL drop of the surfactant sample was placed on a pedestal inside an environmentally controlled chamber set to 37 °C. Following 2 min of adsorption, a computer-controlled stepper motor allowed for dynamic compression and expansion of the drop, reflecting exhalation and inhalation. In total, 20 compression and expansion cycles were performed per sample, with an area compression of approximately 30% and a frequency of 1.5 s per cycle. Images of the surfactant drop were captured at a frequency of 15 frames per second as it underwent dynamic cycling. The images were subsequently analyzed using the Axisymmetric Drop Shape Analysis (ADSA) software, which determined surface tensions reached by the samples based on the shape and area of the drop [22]. Data obtained from the ADSA software was further analyzed in Excel (Microsoft corporation, Redmond, Washington, United States) to produce isotherms displaying the representative surface tension versus relative area for each compression/expansion cycle of the surfactant samples.

### Statistical analysis

Normality of the data was explored using Shapiro–Wilk’s test of Normality and Normal Q-Q plots. Statistical comparisons of clinical variables between groups were performed using independent samples T-test (normally distributed values) or Mann–Whitney test (non-normally distributed values) (IBM SPSS Statistics version 29.0.1.0, SPSS Inc., Chicago, Illinois, United States). Comparisons over consecutive compression expansion cycles were obtained using repeated measures ANOVA and comparisons of single values between groups were analyzed with a T-test. The data was expressed in GraphPad Prism 9 (Dotmatics Inc., Boston, Massachusetts, United States) shown as mean ± standard error of the mean (SEM). P-values ≤ 0.05 were considered statistically significant.

### Ethical approval and owner consent

This study was approved by Viikki Campus Research Ethics Committee, University of Helsinki (Statement 11/2022 1.6.2022). Blood and BALF sampling from healthy dogs were approved by the Board of Animal Experimentation of the Regional State Administrative Agency of Southern Finland (decision ESAVI/10906/04.10.07/2017). Written owner consent was obtained from all owners prior to study participation.

## Results

### Dogs

Seven drevers with recurrent hunting associated pulmonary edema and 7 healthy dogs were included in this study. Demographic data is presented in Table 1. Healthy dogs represented different breeds: Labrador retriever (n = 3) and Australian shepherd, Finnish lapphund, Lapponian herder and Belgian shepherd Groendal, one of each.

**Table 1:** Clinical data

	Drevers (n = 7)	Healthy (n = 7)	P -value
	Mean ± SD Median (IQR)	Mean ± SD Median (IQR)	
Age, years	6.6 ± 2.2	2.3 ± 1.9	0.003
Body weight, kg	16.9 ± 1.8	23.4 ± 5.1	0.011
Sex	Male 5/7	Male 3/7	
	Female 2/7	Female 4/7	
Blood hematology			
Total leucocyte count, 10^9^/L	8.0 ± 2.7	8.1 ± 2.0	n.s
Segmented neutrophils, 10^9^/L	5.4 ± 2.0	5.1 ± 1.7	n.s
Lymphocytes, 10^9^/L	1.6 ± 0.6	2.1 ± 0.7	n.s
Monocytes, 10^9^/L	0.4 ± 0.1	0.3 ± 0.1	n.s
Eosinophils, 10^9^/L	0.5 ± 0.3	0.5 ± 0.3	n.s
Serum biochemistry			
Total protein, g/L	61.6 ± 3.5	57.5 ± 2.8	0.032
Albumin, g/L	31.9 ± 0.9	30.8 ± 1.6	n.s
C-reactive protein, g/L	5.0 (5.0–10.9)	5.0 (5.0–5.0)	n.s

*SD* Standard deviation, *IQR* interquartile range

Demographic data, blood hematology and serum biochemistry results in drever dogs with hunting associated with pulmonary edema (n = 7) and in healthy dogs (n = 7)

In affected drevers the clinical signs had started at a median age of 2 years of age (IQR 2–4 years, range 1–6 years) and at the time of clinical investigations all dogs had experienced several episodes of hunting associated respiratory distress (≥ 10 episodes in 4/7 dogs; 8, 4 and 3 episodes each in one dog). The clinical signs comprised tachypnea (7/7), dyspnea (4/7) and cough (4/7). In 2/7 dogs, clinical signs occurred every time the dog was allowed to hunt, and in 5/7 dogs only after strenuous hunting. None of the owners reported development of respiratory distress, unless the dog found game and was chasing while barking. According to owners, respiratory distress lasted from 6 h up to 72 h after hunting.

**Fig. 1:**
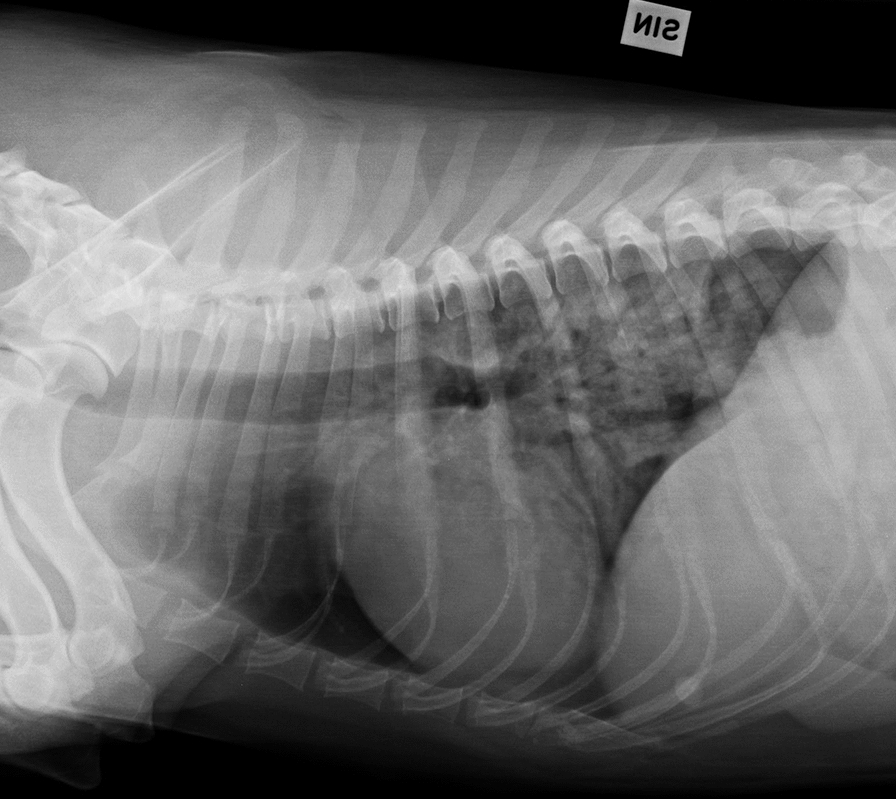
Chest radiograph of a female drever dog during an episode of hunting associated respiratory distress. Latero-lateral chest radiograph showing caudo-dorsal alveolar pattern indicative of pulmonary edema in an affected female drever dog. The radiograph was obtained during an episode of hunting associated respiratory distress, when the dog was 4.5 years old

Thoracic radiographs were obtained during respiratory distress at the referring veterinarians in 5/7 dogs (Fig. 1) and video material describing clinical signs was available for 5/7 dogs. Detailed description of clinical signs as well as video assessment and thoracic radiograph findings for each dog is included as supplementary material (Additional file 1). During veterinary visits, dogs received furosemide and supplementary oxygen. However, all owners reported spontaneous recovery without treatment from subsequent episodes of hunting associated respiratory distress.

All dogs were clinically normal in between episodes, did not have any respiratory signs and had normal exercise tolerance. None of the dogs were receiving medications for hunting associated respiratory distress or any other condition.

### Clinical findings

Cardiorespiratory abnormalities were not detected in any of the dogs during general examination. Results from blood hematology and selected serum biochemistry variables are presented in Table 1. Fecal analysis was performed in all affected dogs with negative results.

Echocardiography examination did not reveal findings indicative of left sided cardiac congestion: La:Ao (mean 1.26, range 1.10–1.39), left ventricular internal diameter end diastole (mean 3.48 cm, range 3.00–4.10 cm), left ventricular internal diameter (2.32 cm, range 1.7–2.70 cm) end systole and fractional shortening (33.3%, range 25–46%) were normal in all affected dogs [23]. No mitral valve regurgitation was documented in 5/7 dogs and a mild mitral valve regurgitation with normal cardiac dimensions was detected in two dogs.

Laryngeal evaluation revealed normal laryngeal function and structure in all affected dogs. In 4/7 dogs a doxapram stimulation (0.5–1.0 mg/kg iv) was necessary to establish normal laryngeal function. Bronchoscopy was performed and respiratory samples were retrieved using BAL in all dogs. Bronchoscopy revealed normal tracheal and bronchial structure in all dogs, bronchial mucosa was considered normal in 4/7 affected dogs and in all healthy controls and mild bronchial mucosal irregularity was detected in 3/7 affected dogs. Results of BALF cytology are presented in Table 2. Bacterial culture was negative in 6/7 affected dogs and in 7/7 healthy dogs. In one affected dog BALF culture yielded small quantity (10^2^ cfu/ml) of mixed growth in the absence of inflammatory cytology interpreted as contamination.

**Table 2:** Bronchoalveolar lavage fluid and surfactant analysis

	Drevers (n = 7)	Healthy (n = 7)	P -value
	Mean ± SD Median (IQR)	Mean ± SD Median (IQR)	
Respiratory cytology			
Total cell count,10^9^/L	0.216 ± 0.127	0.183 ± 0.078	n.s
Neutrophils, %	1.7 ± 0.7	6.8 ± 3.0	0.003
Eosinophils, %	6.0 (1.5–7.5)	6.0 (3.8–21.8)	n.s
Mast cells, %	1.7 ± 1.2	0.5 ± 0.5	0.014
Lymphocytes, %	12.1 ± 12.1	14.2 ± 6.7	n.s
Macrophages, %	73.2 ± 13.2	58.7 ± 17.8	n.s
Surfactant analysis			
BAL phospholipid (µg)	117 ± 19	178 ± 21	n.s
BAL protein (µg/ml)	125 ± 23	123 ± 25	n.s
Adsorption (mN/m)	22.2 ± 0.4	21.9 ± 0.3	n.s
Max surface tension, all cycles (mN/m)	30.8 ± 1.0	29.2 ± 2.2	n.s
Area compression to minimum surface tension, all cycles (%)	27.2 ± 1.7	26.8 ± 1.8	n.s

Bronchoalveolar lavage (BAL) fluid cytology and surfactant analysis results in drever dogs with hunting associated pulmonary edema (n = 7) and in healthy dogs (n = 7)

*SD* Standard deviation, *IQR* interquartile range, *n.s* not significant

Computed tomography imaging did not reveal findings indicative of mechanical upper airway obstruction or other intra- or extra-thoracic abnormalities explaining recurrent hunting associated respiratory distress. None of the dogs had increased lung attenuation indicative of pulmonary edema.

### Surfactant analysis

**Fig. 2:**
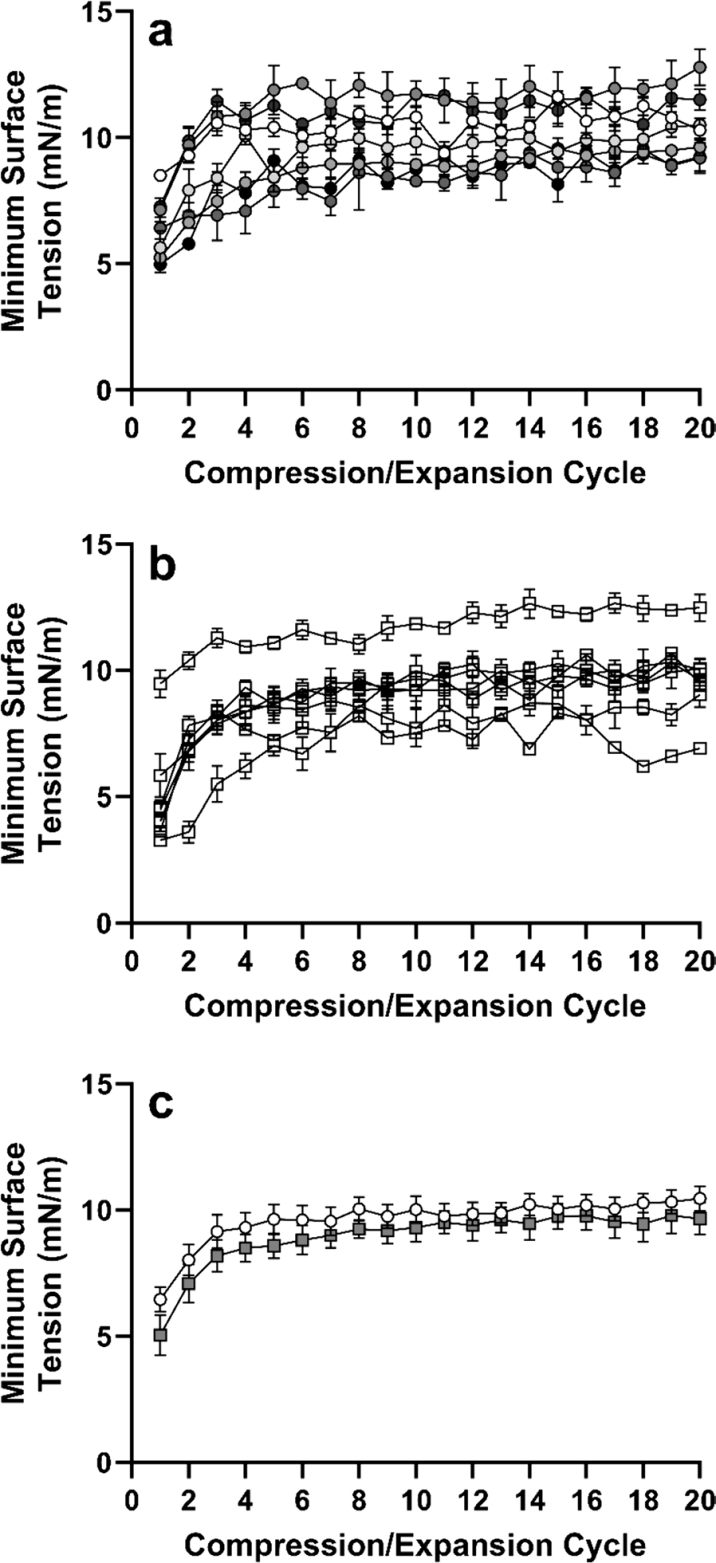
Biophysical properties of alveolar surfactant. Biophysical analysis of the surfactant samples from drever dogs with hunting associated pulmonary edema (n = 7) and from healthy dogs (n = 7). Analysis was performed using a constrained sessile drop surfactometer. The top panel (**A**) shows the minimum surface tensions values obtained from samples from the individual healthy dogs, whereas the middle panel (**B**) shows the data for the individual affected drevers. The average values for the two groups (healthy dogs illustrated with circles, affected drevers illustrated with squares) were not significantly different at any cycle (Panel **C**)

To assess the surfactant activity the isolated samples were analyzed on a constrained sessile drop surfactometer. Shown in Fig. 2 are the minimum surface tensions obtained over 20 expansion/compression cycles. Figure 2a shows the minimum surface tension values obtained from samples from the individual healthy dogs, whereas Fig. 2b shows the data for the affected animals. Although some variability is observed among animals, minimum surface tension generally is a bit lower during the first compression and subsequently remains very consistent between approximately 5 and 12mN/m. The average values for the two groups were not significantly different at any cycle (Fig. 2c). In addition, other biophysical measures, such as the surface tension after 2 min of adsorption and maximum surface tension during compression and expansion cycles were not significantly different between the two groups (Table 2).

## Discussion

To test our hypothesis that the etiology of hunting associated pulmonary edema in drever dogs is associated with impaired biophysical properties, the surfactant from healthy and affected dogs was tested on a constrained sessile surfactometer. The results obtained from 7 healthy and 7 affected animals showed no significant differences in any of biophysical outcomes. Most notable, the critical function of surfactant, reducing the surface tension to low values upon compression, was similar between the two groups. It is concluded that the etiology of this syndrome is not due to an underlying surfactant dysfunction.

An important aspect of our study was the testing of surfactant from affected animals at a time-point at which they did not have any clinical symptoms. Similar protein values in the BAL fluid also indicated that there was no edema at the time of sampling. As such our experiment only tested whether subclinical surfactant dysfunction may contribute to the development of the disease. Considering the literature on the impact of plasma proteins on surfactant function, it is likely that during a clinical episode of hunting associated pulmonary edema, surfactant inhibition may occur and contribute to lung dysfunction [24–27]. As such surfactant dysfunction may still contribute to the pathophysiology of the syndrome, however it appears unlikely to be a contributing factor in the initiation of the edema and clinical signs.

The etiology of hunting associated pulmonary edema in drevers remains yet unclear. It is equally undiscovered whether hunting associated pulmonary edema is mainly due to increased hydrostatic pressure or increased vascular permeability, or both. Measuring edema fluid protein to plasma protein ratio could aid in the future to differentiate high-permeability edema versus hydrostatic edema [5]. Neurogenic mechanisms may be responsible for the development of pulmonary edema in hunting dogs. The pathophysiology of neurogenic pulmonary edema is not fully elucidated, though mechanisms involving hypoglycemia, hypoxemia and fulminant sympathetic stimulation are proposed [5]. It has been suggested that severe sympathetic drive due to strenuous exercise and excitement during hunting may explain the development of pulmonary edema in these dogs [2]. Indeed, it has been shown that excess adrenergic stimulation increases both capillary hydrostatic pressure and capillary permeability, potentially resulting in increased fluid leakage [28, 29]. In addition to the physiological changes favoring edema formation during strenuous exercise, the continuous barking in these hunting dogs may introduce dynamic upper airway obstruction, which further can facilitate the development of pulmonary edema [5]. This is supported by the observation in our study, that respiratory distress occurred in these dogs only when barking while chasing game. However, the important question remains: Why do some individual drever dogs develop marked and long-lasting respiratory distress associated with hunting, while others exposed to equally strenuous hunting remain free of clinical signs? Future studies may continue to search for predisposing factors in affected dogs, for example in dysfunction of the pulmonary safety mechanisms opposing edema formation, such as lung interstitial glycosaminoglycans and pulmonary lymphatics [6, 30]. Additionally, it could be considered whether this syndrome in drevers is similar to exercise induced capillary stress failure and pulmonary hemorrhage in equine athletes; Clinical signs are noted only in a small proportion of horses, but as a matter of fact, most equine athletes are affected to some degree but remain free of obvious clinical signs [31].

Although our study focused solely on the syndrome in drever dogs, it is interesting to note, that exercise induced pulmonary edema is documented to occur also in humans during strenuous exercise such as marathon running. Interstitial lung edema appears to occur relatively commonly and has been documented in 17–46% of marathon finishers [32–35]. In most cases the clinical significance of this interstitial edema appears to be minor, although sporadic cases of severe alveolar edema and dyspnea have also been reported [33, 35–37]. The etiology of this phenomenon is ambiguous and marked interindividual variety in the proneness to develop edema has been documented in humans [6, 7]. Whether or not the findings in humans are related to the syndrome in drever dogs will require further studies.

Limitations of the current study include the small number of dogs in each group and the lack of a control group consisting of healthy drevers; as the control group comprised various breeds, there was a statistically significant age and weight difference when compared to affected drevers. However, this was considered clinically less significant as both groups represented medium sized adult dogs. Additionally, ethical considerations favored collecting BALF samples in dogs already anesthetized for another procedure, rather than inducing anaesthesia in healthy drevers solely for research purposes. Affected drevers in our study had significantly less neutrophils in their BALF compared to the control group. This could represent a decreased breed related tendency to airway inflammation, or it may represent a difference in the environmental conditions the dogs were living: The affected drevers lived in the countryside with less air pollution and particles compared to the control dogs mostly living in urban areas [38, 39]. Another limitation is, that thoracic radiographs were not obtained during a previous episode of hunting associated respiratory distress in 2/7 affected drevers and therefore it cannot be fully confirmed, that findings indicative of pulmonary edema were present. However, in both dogs, the clinical picture with recurrent episodes of hunting associated respiratory distress resolving without specific treatment, video material showing expiratory dyspnea, as well as extensive examinations excluding other cardiopulmonary conditions was considered highly indicative of hunting associated pulmonary edema prevalent in this breed. Furthermore, in this study, cell-free BALF samples were frozen prior to isolation of surfactant and analysis, which is also a limitation. In other studies, the BALF is centrifuged to isolate only the active subfraction of surfactant, the so-called large aggregates, whereas with our methodology a combination of large and small aggregates were isolated [40, 41]. Thus, the biophysical analysis reflects the combined results of the two subfractions. Had we observed differences between the two groups, this limitation could have affected our interpretation of the data with respect to the impact of the relative amounts of the different subfractions. However, considering the lack of differences between our two groups, this methodological difference did not affect our conclusion.

## Conclusions

Our study showed that the biophysical ability of alveolar surfactant to reduce surface tension was similar in drevers with hunting associated pulmonary edema and in healthy dogs of other breeds. These findings indicate that the etiology of hunting associated pulmonary edema in drever dogs is not due to an underlying surfactant dysfunction.

### Supplementary Information

Additional file1: Demographic and clinical characteristics of drever dogs with hunting associated respiratory distress.

## Data Availability

The datasets used and analyzed during the current study are available from the corresponding author upon a reasonable request.

## Abbreviations

ADSA: Axisymmetric Drop Shape Analysis
BAL: Bronchoalveolar lavage
BALF: Bronchoalveolar lavage fluid
CT: Computed tomography
IQR: Interquartile range
LA:Ao: Left atrium-aorta ratio
