# Supplementary material for: Biophysical properties of alveolar surfactant in drever dogs with hunting associated pulmonary edema
Source: Acta Vet Scand. 2024 May 31;66:24. doi: 10.1186/s13028-024-00745-x (PMC11143697; doi:10.1186/s13028-024-00745-x)
Supplement: Supplementary file 1 — Additional file1: Demographic and clinical characteristics of drever dogs with hunting associated respiratory distress. [file 13028_2024_745_MOESM1_ESM.pdf]

Additional file 1: Demographic and clinical characteristics of drever dogs with hunting associated respiratory distress (n=7)

No.	Sex	Age (years) at the time of examinations	Age (years) at the onset of clinical signs	Number of previous hunting associated respiratory distress episodes	Duration of respiratory distress (hours) estimated by the owners	Clinical signs described by the owner	Thoracic radiographs obtained during respiratory distress	Radiographic findings	Video assessment by a pulmonologist	Respiratory type<br>Respiratory rate<br>Other findings<br><br>Assessed from video recordings
1	M	6,9	2	10	6-24	Tachypnea<br>Dyspnea	No		Yes	Expiratory dyspnea<br>RR72/min<br>Lying on ventral recumbency, mouth closed, extended neck, elevated nose
2	M	5,5	1	>10	6-10	Tachypnea<br>Dyspnea<br>Cough	No		Yes	Expiratory dyspnea<br>RR 54/min<br>Productive coughing<br>Standing / sitting, mouth closed
3	M	5,5	2	8	24-72	Tachypnea	Yes	Perihilar alveolar and caudo-dorsal interstitial pattern<br>Small local area of cranio-ventral alveolar pattern<br>Mild pleural effusion	No	Expiratory dyspnea<br>RR 112/min<br>Lying on lateral recumbency, mouth closed, extended neck
4	F	6,6	3	>10	10-24	Tachypnea<br>Cough	Yes	Caudo-dorsal alveolar pattern	Yes	Expiratory dyspnea<br>RR 64/min<br>Standing, mouth closed, extended neck
5	M	4,0	2	>10	12-24	Tachypnea<br>Cough	Yes	Caudo-dorsal patchy alveolar and interstitial pattern	Yes	Expiratory dyspnea<br>RR 82/min<br>Standing, mouth closed, extended neck
6	MN	10,0	4	4	24	Tachypnea<br>Dyspnea<br>Cough	Yes	Interpreted as pulmonary edema by the referring veterinarian.<br><br>Images not available for review	No	
7	F	9,6	6	3	12-24	Tachypnea<br>Dyspnea	Yes<br>(two separate occasions)	Perihilar and caudo-dorsal alveolar and interstitial pattern	No	

M= male, F= female, MN= male neutered, RR= respiratory rate
